# Comparison of purse-string suture versus over-the-scope clip for gastric endoscopic full-thickness closure: traction and leak pressure testing in ex vivo porcine model

**DOI:** 10.1186/s12893-023-01920-z

**Published:** 2023-01-26

**Authors:** Takanori Matsui, Hideki Kobara, Noriko Nishiyama, Kaho Nakatani, Tingting Shi, Naoya Tada, Kazuhiro Kozuka, Nobuya Kobayashi, Taiga Chiyo, Tatsuo Yachida, Akihiro Kondo, Takayoshi Kishino, Keiichi Okano, Shintaro Fujihara, Kunihisa Uchita, Kingo Hirasawa, Tsutomu Masaki

**Affiliations:** 1 Department of Gastroenterology and Neurology, Faculty of Medicine, Kagawa University, 1750-1 Ikenobe, Kita, Miki, Kagawa 761-0793 Japan; 2 Department of Gastroenterological Surgery, Faculty of Medicine, Kagawa University, Kita, Japan; 3 Department of Gastroenterology, Kochi Red Cross Hospital, Kochi, Japan; 4 Division of Endoscopy, Yokohama City University Medical Center, Yokohama, Japan

**Keywords:** Endoscopic closure, Endoscopic full-thickness resection, Purse-string suture, Over-the-scope clip

## Abstract

**Background:**

The recently developed endoscopic full-thickness resection technique requires reliable closure. The main closure methods are the purse-string suture (PSS) technique and over-the-scope clip (OTSC) technique; however, basic data on the closure strength of each technique are lacking. This study was performed to compare the closure strengths of these two methods in an ex vivo porcine model.

**Methods:**

In the traction test, a virtual 5-cm full-thickness closure line was closed by the following six methods three times each: conventional hemoclips, mucosal PSS, seromuscular PSS, mucosal OTSC, seromuscular OTSC, and surgical suture. The primary endpoint was the tension at the starting point of dehiscence, measured in Newtons (N) by an automatic traction machine. In the leak test, a 15-mm gastric full-thickness defect was closed by PSS or OTSC six times each, and the closed stomach was then pressurized in a water container. The primary endpoint was the leak pressure when air bubbles appeared. The secondary endpoints were the procedure time and presence of complete inverted closure.

**Results:**

The mean tension was 2.16, 3.68, 5.15, 18.30, 19.30, and 62.40 N for conventional hemoclips, mucosal PSS, seromuscular PSS, mucosal OTSC, seromuscular OTSC, and surgical suture, respectively. Complete inverted closure was observed for seromuscular PSS, seromuscular OTSC, and surgical suture. The mean leak pressure was 13.7 and 24.8 mmHg in the PSS and OTSC group, respectively (P < 0.01). The mean procedure time was 541 and 169 s in the PSS and OTSC group, respectively (P < 0.01). Complete inverted closure was observed in OTSC alone.

**Conclusion:**

The OTSC, which allows complete inverted closure, showed greater closure strength than PSS. Considering the size limitation suitable for single OTSC, a therapeutic strategy for closing the larger size is further warranted.

## Introduction

Gastrointestinal stromal tumors (GISTs) are the most common mesenchymal tumors of the gastrointestinal tract [1], with a reported incidence of 10 to 15 per million people per year [2]. The stomach is the most common site of GISTs, accounting for 55.6% of cases [2]. The 5-year survival rate in patients with GISTs is 83%, and this rate increases to 93% when GISTs are organ-confined [3]. Therefore, it is important to treat GISTs when they are organ-confined.

Surgical intervention is the first therapeutic option for resectable GISTs, and partial gastrectomy or gastric wedge resection is the standard of care [4]. Laparoscopic and endoscopic cooperative surgery emerged in 2008 [5] as a minimally invasive treatment for GISTs. Because of its high reported efficacy and safety [6], this procedure has been widely performed in Japan and around the world.

In 2001, endoscopic full-thickness resection (EFTR) was developed to achieve complete resection of gastrointestinal neuroendocrine tumors and defect closure using only a flexible endoscope [7]. EFTR without laparoscopic assistance has several advantages over conventional approaches. No scar occurs on the skin surface, leading to a reduction in patient complaints. Furthermore, EFTR had shorter operative time and less blood loss [8, 9]. Additionally, the nerves around the stomach are preserved, preventing postoperative gastric motility disorder [9, 10]. Thus, EFTR is considered a more minimally invasive procedure. Although EFTR has already been clinically introduced in some countries [11, 12], several issues remain unresolved [13]. The most important of these issues is reliable endoscopic closure of the full-thickness defect after EFTR. Major endoscopic closure methods include the use of hemoclips [14], the purse-string suture method (PSS) [15], and the over-the-scope clip (OTSC) [16, 17], but sufficient evidence regarding these closure methods during EFTR is lacking. A previous study showed that the mean leak pressures at the endoscopic gastric closure sites were 32.5 mmHg for 5-mm defects, 111.9 mmHg for 10-mm defects, 74.9 mmHg for 15-mm defects, 49.3 mmHg for 20-mm defects, and 15.2 mmHg for 25-mm defects, indicating that the closure pressure decreased as the defect size increased [18]. Accordingly, the closure strength of each method must be compared to establish the most appropriate closure technique. However, few reports have examined and compared the closure strength of these closure methods [18, 19].

This study was performed to examine the closure strengths of PSS and OTSC in an ex vivo porcine model and explore a closure method suitable for EFTR.

## Methods

This ex vivo study involved 18 porcine stomachs isolated from pigs used for food. Fresh ex vivo stomachs were harvested from mixed-breed pigs weighing 100 to 120 kg at 6 months of age (Tokyo Shibaura Zouki, Tokyo, Japan). These pigs were raised on farms in Japan and euthanized for food while unconscious from CO_2_. The porcine stomachs were frozen and thawed immediately before use. We then washed the insides of the stomachs with tap water. A flexible endoscope and endoscopic instruments were prepared for performance of each endoscopic closure method. The ex vivo study consisted of traction and air leak pressure tests to evaluate the closure strength of each closure method for EFTR. To minimize selection bias between operators, endoscopic clip closure was performed by N.N. only. Surgical closure was performed by A.K. and T.K. Finally, all obtained data were compared among the closure methods.

### Traction test

#### Study protocol

**Fig. 1:**
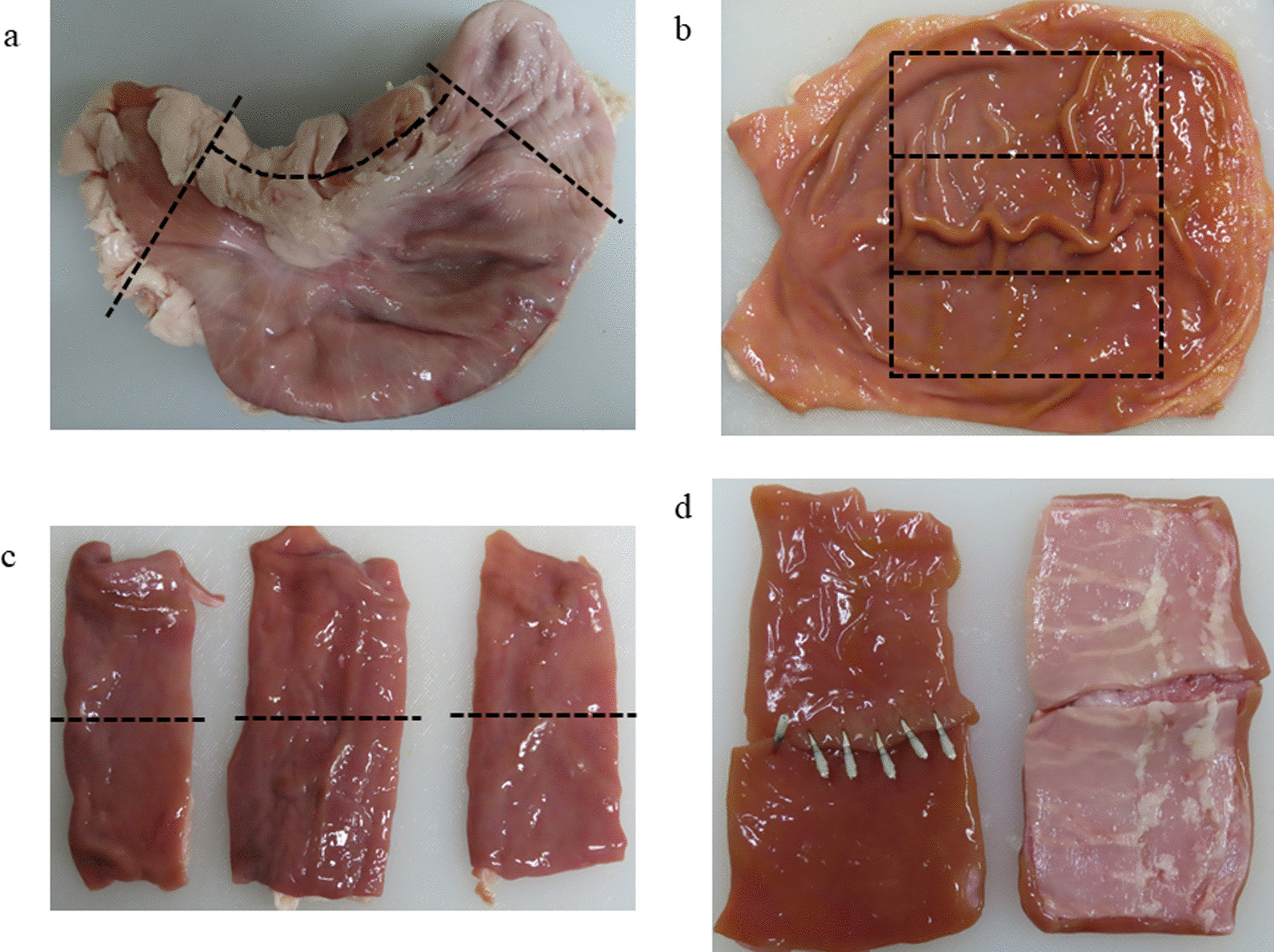
Procedure for creating full-thickness specimens of porcine stomach. **a** The porcine stomach was openly incised on the lesser curvature side (black lines). **b** Three specimens of 8-cm length and 5-cm width were excised from the greater curvature of the body of the stomach (surrounding black). **c** The full-thickness layers were disconnected at the center of the 8-cm-long side of the specimen, shown as dehiscence lines (black lines). **d** The dehiscence line was closed by each closure method. The left figure indicates the mucosal side, and the right figure indicates the serosal side

**Fig. 2:**
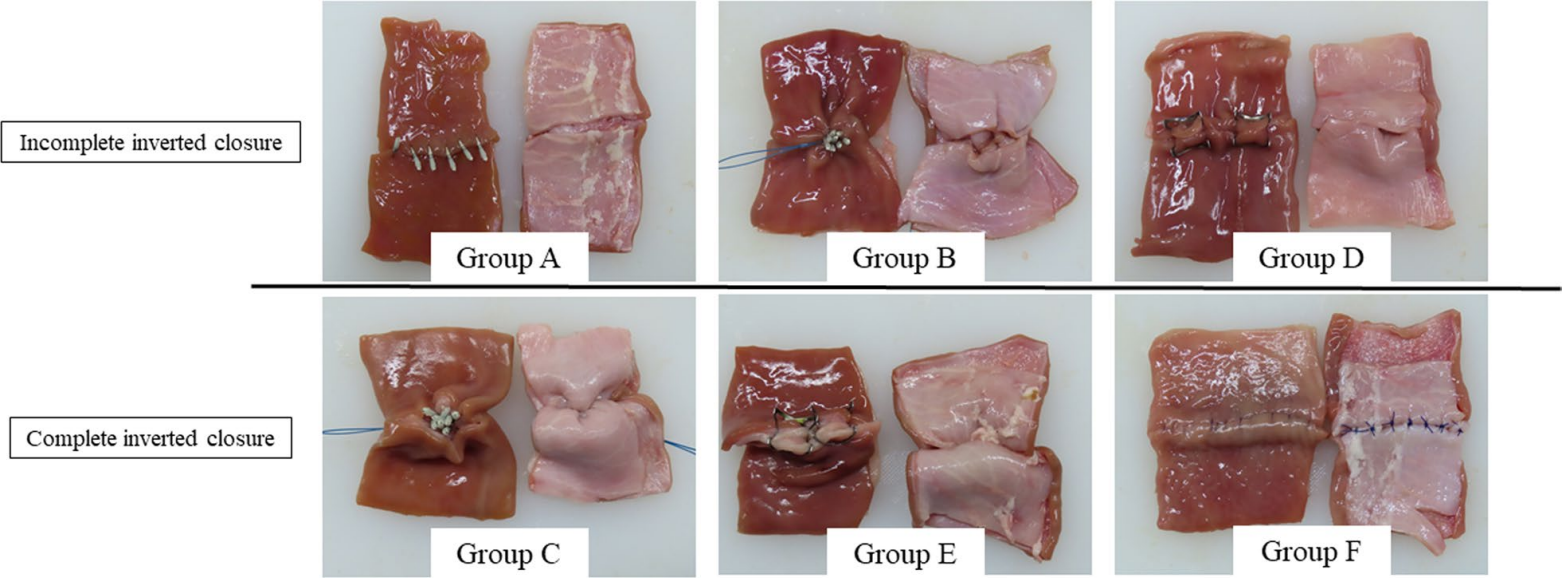
Six closure methods. Left specimens: mucosal side, Right specimens: serosal side in each group. Group **A** hemoclip closure, Group **B** purse-string suture (PSS) (mucosal closure), Group **C** PSS (seromuscular closure), Group **D** over-the-scope clip (OTSC) closure (mucosal closure), Group **E** OTSC closure (seromuscular closure), Group **F** surgical hand suture. Upper section: absence of complete inverted closure in Groups **A**, **B**, and **D**. Lower section: presence of complete inverted closure in Groups **C**, **E**, and **F**

Six porcine stomachs were prepared, and for each porcine stomach, three specimens of 8-cm length and 5-cm width were excised from the greater curvature of the body of the stomach, resulting in a total of 18 specimens. The full-thickness layers were disconnected at the center of the 8-cm-long side of the specimen, and the dehiscence line was closed by each closure method. Each stomach was closed using the same method for each of the three specimens (Fig. 1). All closure methods using endoscopic instruments were performed using a flexible endoscope. The closure methods were categorized into the six methods described below and shown in Fig. 2.

#### Endoscopic procedures

##### Group A: Hemoclip closure (n = 3)

Hemoclips (HX-610–090; Olympus, Tokyo, Japan) were used to close the dehiscence line at 7-mm intervals.

##### Group B: PSS (mucosal closure) (n = 3)

A detachable endoloop (MAJ-254; Olympus) was placed to cover both sides of the dehiscence line. After fixing it to the mucosal surface with eight hemoclips at 7-mm intervals, the dehiscence line was closed while tightening the endoloop.

##### Group C: PSS (seromuscular closure) (n = 3)

After anchoring the endoloop on both the serosa and muscle using hemoclips, the above-described PSS method was applied.

##### Group D: OTSC closure (mucosal closure) (n = 3)

A 12-mm OTSC (12-gc type; Ovesco Endoscopy AG, Tübingen, Germany) [17] was mounted on the tip of the endoscope (GIF-260QJ; Olympus). After only the mucosa of both edges on the dehiscence line was grasped with Twin Grasper forceps (TG forceps) (Ovesco Endoscopy AG), an OTSC was fired while pulling the TG forceps into the cap. Two OTSCs were used to close the 5-cm dehiscence line.

##### Group E: OTSC closure (seromuscular closure) (n = 3)

After the serosa as well as the muscle layers of both edges on the dehiscence line were grasped with the TG forceps, the above-described OTSC closure technique was used.

##### Group F: Surgical hand suture (n = 3)

Using a 3–0 surgical nylon thread, the surgeon sutured the 5-cm dehiscence line from the serosal side with Albert-Lembert sutures at 7-mm intervals as shown in the appearance of thread knots on the serosal side (Fig. 2).

#### Mechanical measurement

**Fig. 3:**
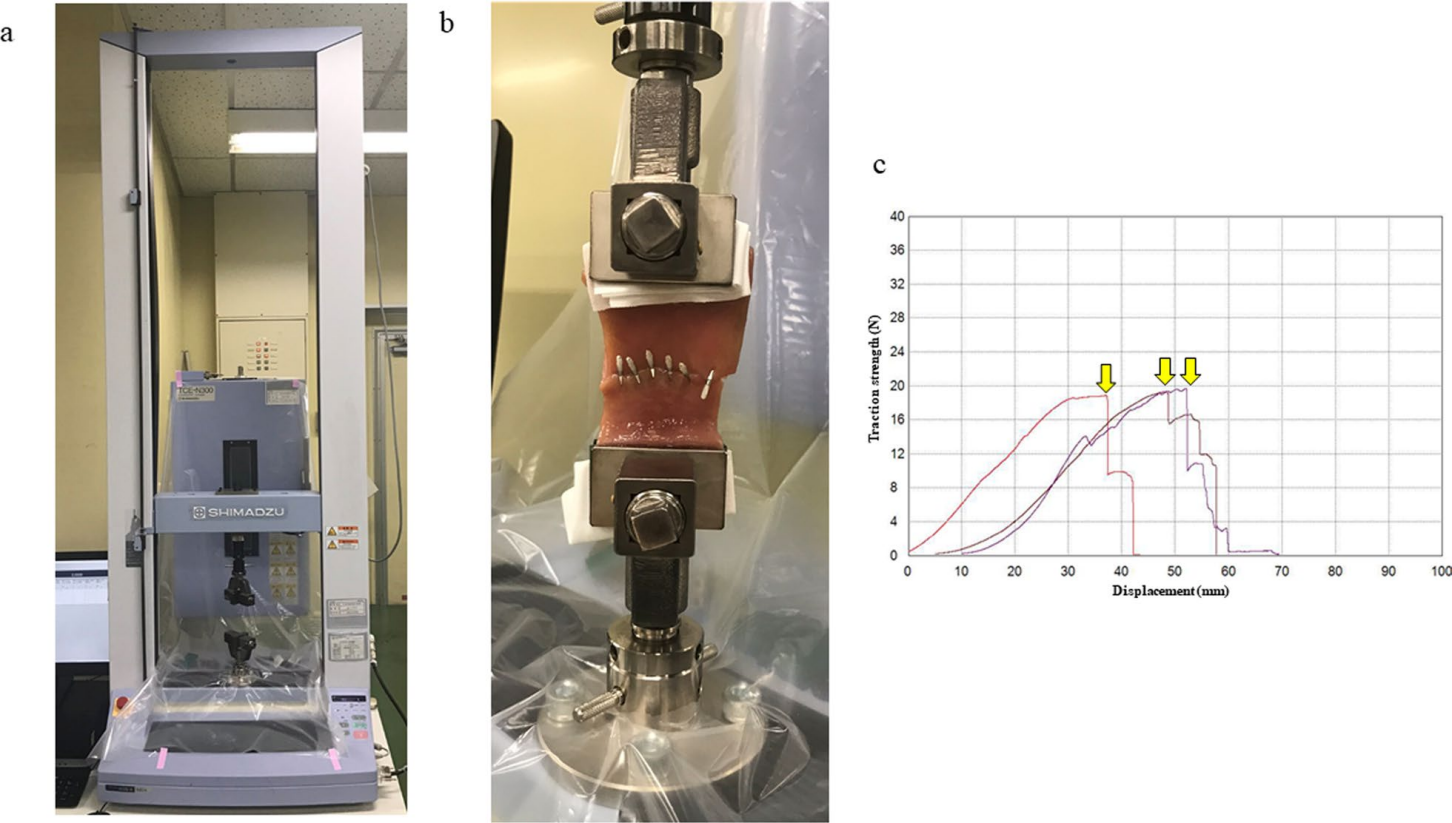
Measurements of traction strength. **a** Traction machine (Autograph; Shimadzu Corporation, Kyoto, Japan). **b** Both sides of each closed specimen were fixed on the lower and upper arms. The upper arm automatically pulled the specimen toward the upper direction at the speed of 1 mm/s. **c** Computer-generated waveform. Traction strength was measured in Newtons (N) when the dehiscence began, which correlated with the top of the first waveform

A traction machine (Autograph; Shimadzu Corporation, Kyoto, Japan) was used to measure the traction strength. Both sides of each closed specimen were fixed on the lower and upper arms. The upper arm automatically pulled the specimen toward the upper direction at the speed of 1 mm/s (Fig. 3). The traction strength was measured in Newtons (N) based on the computer-generated waveform.

#### Outcome measures

The primary endpoint was comparison of the tension when the dehiscence began, which correlated with the top of the first waveform. The secondary endpoint was the presence or absence of complete inverted closure on the serosal surface. Complete inverted closure was defined as inversion of the whole closure line. The presence of complete inverted closure was evaluated by three specialized endoscopists.

### Air leak test

#### Study protocol

A 15-mm × 15-mm full-thickness defect was created with a surgical scalpel from the serosal side in the anterior wall of the gastric upper body. A 15-mm × 15-mm circular paper was used to unify the size of all defects. The duodenum was occluded with surgical nylon to prevent other air leaks. An endoscope was inserted into the stomach via the hole on the esophageal side, and closure was then performed. Twelve porcine stomachs were randomly assigned to two groups for defect closure: the PSS group (n = 6) or OTSC group (n = 6). The defect was endoscopically closed by PSS or OTSC without hand assistance to create ex-vivo setting close to clinical practice. Regarding the accuracy of closure, we believed that 15-mm full-thickness defect could be completely closed based on the previous report [20]. When small perforation after closure was confirmed under the overview of the serosal side, the case was discarded. Finally, all procedures were completed without excluded cases.

#### Endoscopic procedures

In the PSS group (n = 6), an endoloop was placed on the defect. After fixing it onto the full-thickness layer with six hemoclips at 5-mm intervals, the defect was closed while tightening the endoloop. In the OTSC group (n = 6), the full-thickness layers of both edges of the defect were grasped with the TG forceps, and the above-described OTSC closure technique was then performed using a single OTSC.

#### Mechanical measurement

**Fig. 4:**
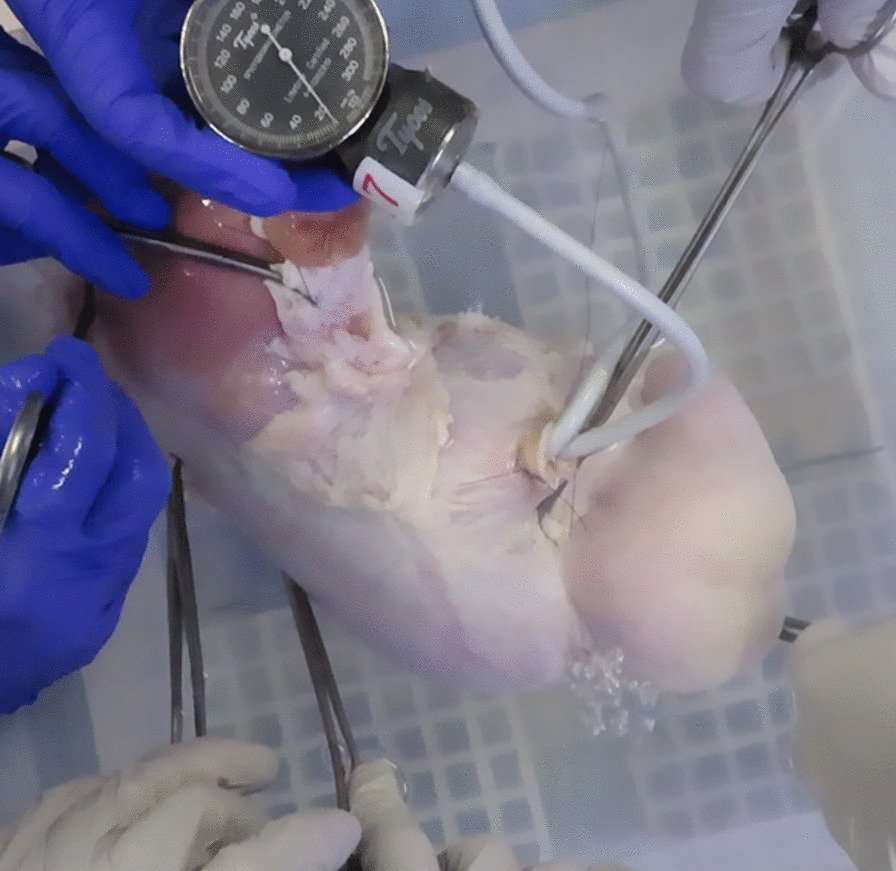
Air leak test. The sealed stomach was inserted into a container filled with water using multiple surgical forceps. The stomach was slowly inflated using the air pump. Finally, the pressure gauge reading was recorded as the leak pressure when air bubbles appeared at the closure site

After completion of the defect closure, two tubes of a blood pressure instrument were inserted into the stomach via the hole on the esophageal side of the endoscopic access route One of the tubes was connected to a pump that could be pressurized to inflate the porcine stomach, and the other tube was connected to a pressure gauge calibrated in millimeters of mercury (mmHg) to measure the pressure in the stomach. The sealed stomach was inserted into a container filled with water using multiple surgical forceps. The stomach was slowly inflated using the air pump. Finally, the pressure gauge reading was recorded as the leak pressure when air bubbles were observed at the closure site (Fig. 4).

#### Outcome measures

The primary endpoint was comparison of the mean leak pressure (mmHg) between PSS and OTSC. The secondary endpoints were the procedure time and presence of complete inverted closure. The procedure time was the duration between the start and completion of each closure method. The criterion for the start was deployment of the endoloop around the defect in the PSS group or the grasping of one side of the defect with the TG forceps in the OTSC group. In both groups, completion of a closure method was defined as the confirmation of complete closure.

### Statistical analyses

All statistical analyses were performed with GraphPad Prism 7.0 (GraphPad Software, San Diego, CA, USA). Comparisons between each group were performed by one-way analysis of variance and the Mann–Whitney U test. A *P*-value of < 0.05 was considered significant. This study did not require IRB approval.

## Results

### Traction test

**Fig. 5:**
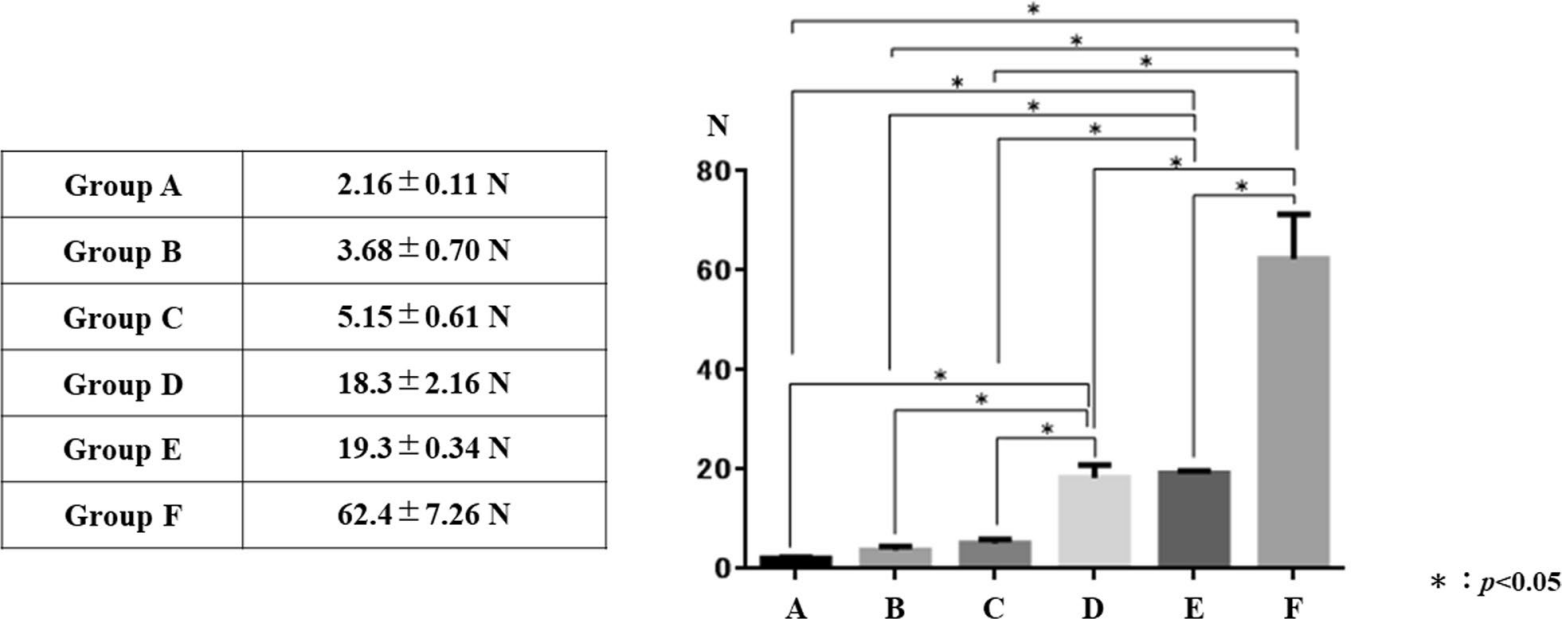
Outcome results of traction test. The mean ± standard deviation traction tension measured for the three samples in each group was 2.16 ± 0.11 N, 3.68 ± 0.70 N, 5.15 ± 0.61 N, 18.30 ± 2.16 N, 19.30 ± 0.34 N, and 62.40 ± 7.26 N for Groups **A**–**F**, respectively. There were no significant differences among Groups **A**, **B**, and **C**. Compared with these three groups, Groups **D**, **E**, and **F** had significantly stronger traction tension. In addition, there was no significant difference between Groups **D** and **E**. However, the traction tension was significantly stronger in Group F than in Groups **D** and **E**

The mean ± standard deviation (SD) traction tension measured for three samples in each group was 2.16 ± 0.11 N, 3.68 ± 0.70 N, 5.15 ± 0.61 N, 18.30 ± 2.16 N, 19.30 ± 0.34 N, and 62.40 ± 7.26 N for Groups A, B, C, D, E, and F, respectively (Fig. 5).

There were no significant differences among Groups A, B, and C. Compared with these three groups, Groups D, E, and F had significantly stronger traction tension. In addition, there was no significant difference between Groups D and E. However, the traction tension was significantly stronger in Group F than in Groups D and E (Fig. 5). Complete inverted closure was observed in Groups C, E, and F (Fig. 2).

### Air leak test

**Fig. 6:**
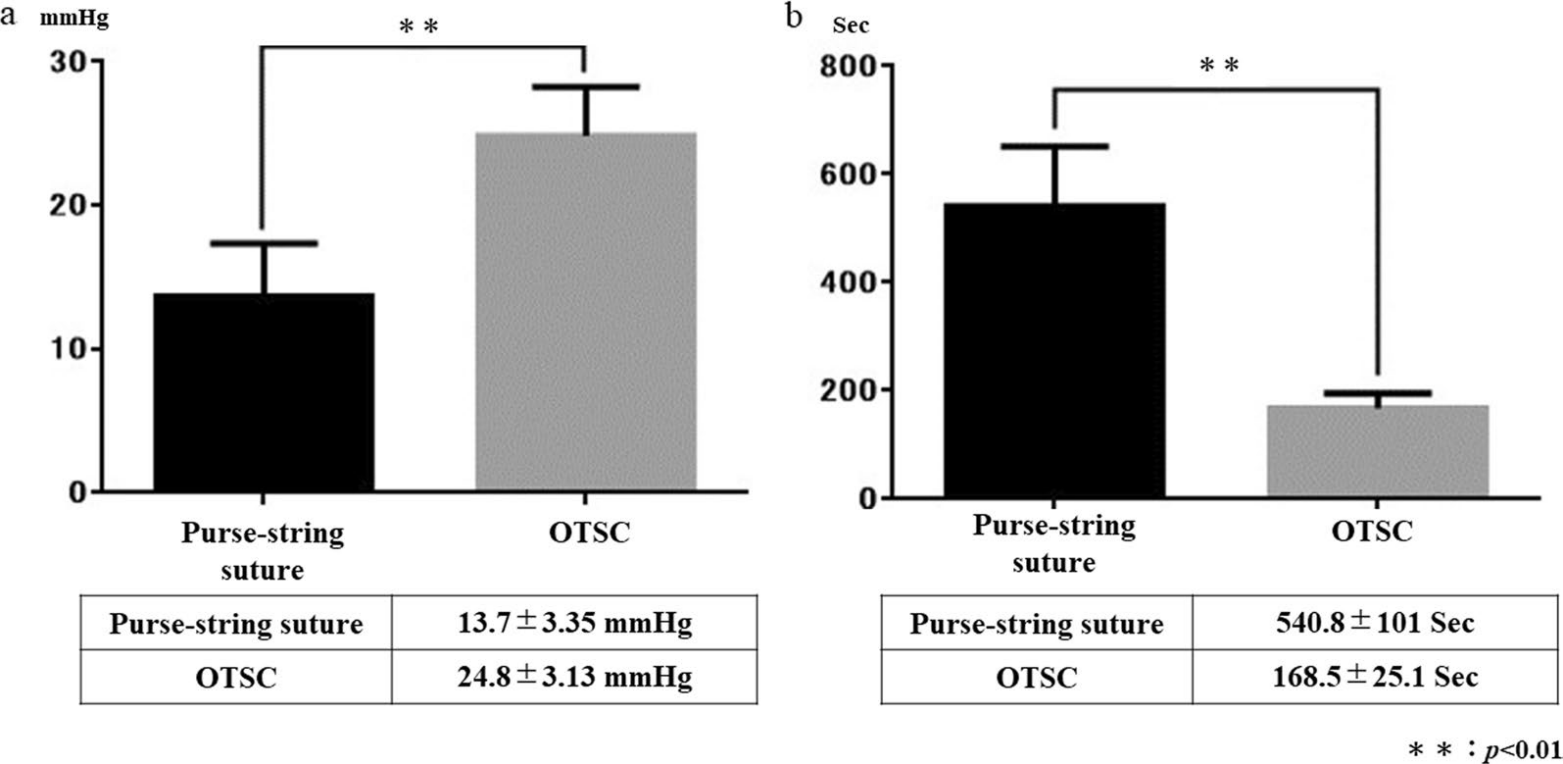
Outcome results of air leak test. **a** The mean ± standard deviation air leak pressure was 13.7 ± 3.35 mmHg in the PSS group and 24.8 ± 3.13 mmHg in the OTSC group (P < 0.01). **b** The mean ± standard deviation procedure time was significantly shorter in the OTSC group than in the PSS group (168.5 ± 25.1 vs. 540.8 ± 101 s, respectively) (P < 0.01)

**Fig. 7:**
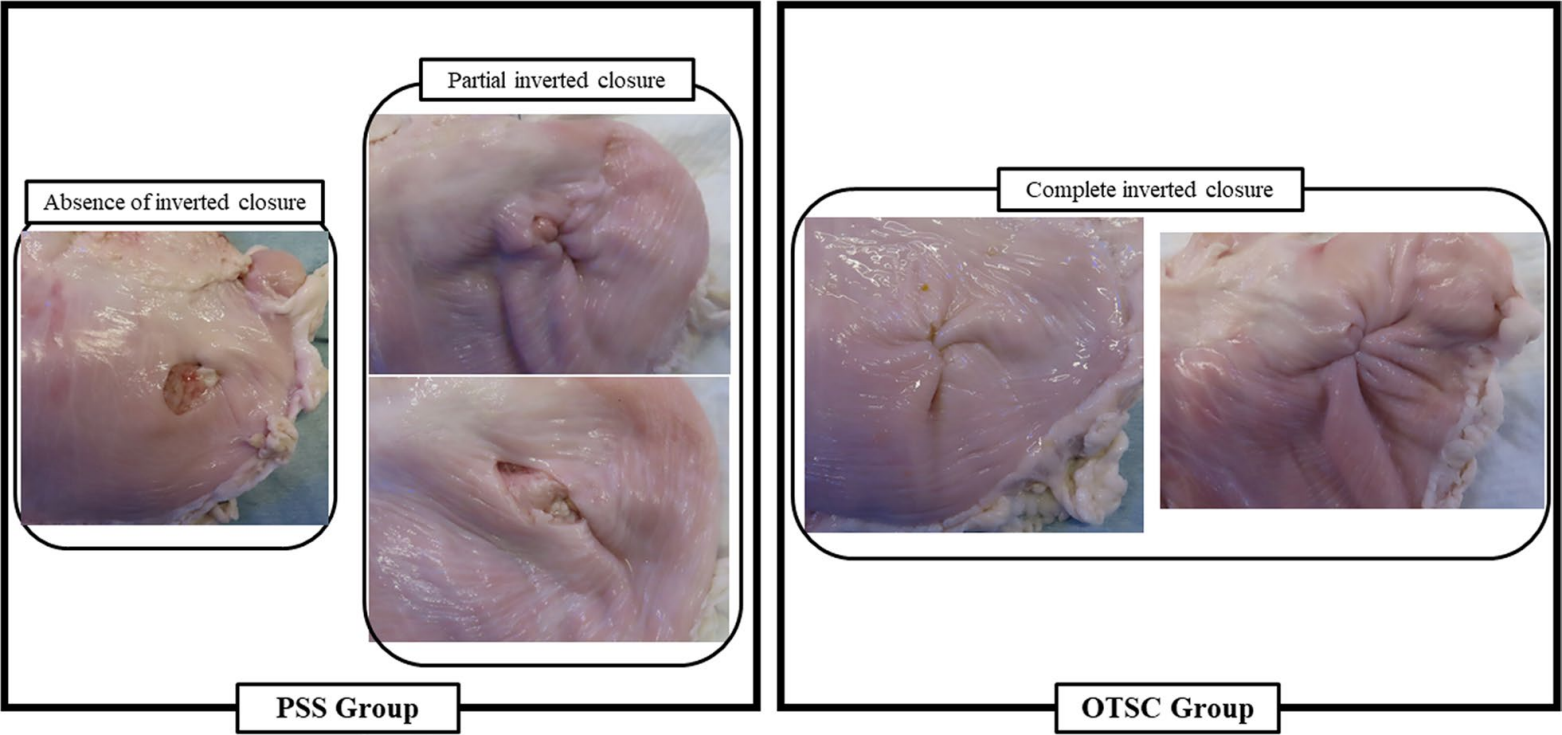
Presence of complete inverted closure in air leak test. Complete inverted closure was observed only in the OTSC group; the PSS group exhibited absence of inverted closure [50% (3/6)] and partial closure [50% (3/6)]

The mean ± SD air leak pressure by the closure method was 13.7 ± 3.35 mmHg in the PSS group and 24.8 ± 3.13 mmHg in the OTSC group (Fig. 6a). In the statistical analysis, the OTSC group showed a significantly higher leak pressure than the PSS group (*P* < 0.01). The mean ± SD procedure time was significantly shorter in the OTSC group than in the PSS group (168.5 ± 25.1 vs. 540.8 ± 101 s, respectively) (Fig. 6b). Complete inverted closure was observed only in the OTSC group; the PSS group showed absence of complete inverted closure [50% (3/6)] as well as partial closure [50% (3/6)] (Fig. 7).

## Discussion

This is the first study to compare the closure strength of endoscopic closure methods for gastric full-thickness defects using traction force and air leak pressure testing. In this basic study, we found that an OTSC suitable for inverted closure produced a higher closure strength than PSS. The surgical suture was also significantly stronger than the other closure methods.

EFTR is still challenging, and several issues regarding its establishment have been raised. Among these issues, the most important is the development of a reliable endoscopic closure method for full-thickness defects. Inadequate closure can cause dehiscence of the closure line, leading to serious complications such as peritonitis and sepsis. Therefore, the closure strength of current closure methods should be fundamentally acknowledged. In our comparative study, we selected conventional hemoclip closure, PSS [21], and OTSC [21], all of which have been reported as the main endoscopic closure methods after EFTR. We then performed two tests, namely mechanical traction and air leak pressure tests, that have been traditionally evaluated in the surgical field.

The traction test showed no significant difference in traction strength between hemoclip closure and PSS; however, the traction strength of OTSC was significantly stronger than that of hemoclip closure and PSS. The traction strength of surgical suture was also significantly stronger than the other closure methods. A recent review of EFTR showed that the rate of convert to surgery was 1.1% in hemoclips, 0.4% in PSS, and 0% in OTSC, respectively [22]. Although hemoclips closure was traditionally introduced, current reports mostly involved PSS and OTSC. Moreover, the hemoclips alone shows clinically technical difficulty for the defect approximation because of luminal collapse caused by the perforation. We therefore selected PSS and OTSC expect for hemoclips closure in the air leak test. The air leak test showed that the intragastric pressure required to cause an air leak was significantly higher in OTSC than in PSS, indicating that OTSC has greater closure strength. The procedure time was shorter in OTSC than in PSS. Whereas anchoring several hemoclips around the endoloop requires a longer time in PSS, TG forceps-assisted OTSC enables closure of a large defect in a single step.

These two tests concluded that the closure strength was greater in the OTSC group than in the PSS group. Meanwhile, both the traction strength and air leak pressure were lower in the OTSC group than in the surgical suture group. In clinical practice, the degree of leak pressure needed for durable closure after EFTR should be discussed. The intragastric pressure during physiological fasting is considered to be 6.6 mmHg [23], and the pressure does not increase even with food intake [24]. The mean leak pressures in this study, which were 13.7 mmHg in the PSS group and 24.8 mmHg in the OTSC group, were higher than the previously reported pressure of 6.6 mmHg. Therefore, both PSS and OTSC may be acceptable means of closing the closure line after EFTR under usual conditions. However, because the intragastric pressure increases with obesity [25] and markedly increases during coughing and vomiting [26], a stronger closure is preferable in these situations.

Whether mucosal closure or seromuscular closure is suitable for full-thickness closure remains unclear. The PSS technique [27] principally anchors the circumferential mucosa around the defect, whereas clip anchoring of the seromuscular layer of both defect edges is expected to be better for inverted closure. In OTSC, the areas grasped by the TG forceps were also divided into mucosa or serosa-muscle. We therefore conducted two patterns of mucosal closure or seromuscular closure. In this comparison in the PSS group, although not significant, the traction strength tended to be slightly stronger in seromuscular closure than in mucosal closure. In the traction test, complete inverted closure of the serosa-muscle layer was observed only in seromuscular closure in both the PSS and OTSC groups. In the air leak test, complete inverted closure was observed only in the OTSC group. The principle of surgical suturing is traditionally based on inverted suturing of the serosa-muscle layer, such as the Albert-Lembert suture technique [28, 29]. However, even if endoscopic closure using hemoclips (as in PSS) appears endoluminally to be complete defect closure, the state of the serosal side is not well known. The present study clarified that mucosal closure does not satisfy the criterion for inverted closure being suitable for surgical suture. Thus, anchoring hemoclips on the serosa-muscle or full-thickness layer seems mandatory in PSS, and grasping these layers with TG forceps is also favorable in OTSC.

A 15-mm defect size may sound small considering the EFTR. However, we considered the two following reasons: Currently, EFTR applies endoscopic submucosal dissection technique to minimize the perforation size because the reliable closure technique for large defect is still under investigation. This procedure comprises submucosal dissection and tumor resection under direct vision. Thus, the defect size is expected to be small size up to 15-mm [22]. Furthermore, a previous report revealed that a diameter up to 15-mm could be completely closed with a single OTSC [20]. If the defect size is more than 15-mm, two OTSC are needed for closure, having a concern of leak in the gap between deployed OTSCs. Consequently, 15-mm defect size was set up according to clinical manner.

A previous ex vivo porcine study showed that gastric OTSC closure of 15-mm full-thickness defects sustained a higher mean (± SD) air leak pressure (74.9 ± 17.5 mmHg) than surgical stapling (64.6 mmHg) [18]. Moreover, gastric OTSC closure of mean 16.29 -mm full-thickness defects sustained a similar air leak pressure (72.5 mmHg) in the porcine stomach in natural orifice transluminal endoscopic surgery (NOTES) [19]. These leak pressures were higher than that obtained in the present study (24.8 mmHg). The difference may be explained by technical aspects, such as pulling the defect into the OTSC cap using TG forceps, as well as the difference in the gastric wall thickness of the porcine models.

A recent meta-analysis revealed that the clinical complication rate of EFTR was 1.6%, including a 0.1% rate of delayed suture dehiscence and a 0.9% rate of intra-abdominal infection [11]. Three studies demonstrated that intra-abdominal infection occurred in either hemoclip closures or PSS, whereas none occurred in OTSC use [11].

This study has five limitations. First, the experiment was conducted using a porcine stomach. The thickness of the mucosa and muscular layer and the expansion and contraction of the gastric wall differ from those of a human stomach. Nevertheless, different endoscopic closure methods were compared under the same conditions to examine the closure strength. Second, the durability and wound healing after suturing were not considered because of the nature of the ex vivo experiments. The healing process may differ depending on the closure method used. In vivo studies are ongoing to investigate this issue. Third, the defect size in the leak test was not substantially large (15-mm). EFTR is indicated for gastric GISTs of ≤ 3 cm; thus, we plan to perform measurement of leak pressure in 3-cm defects. The traction test alone does not investigate leaks. Especially, the gaps between two OTSCs, have a high potential risk of leak. The testing of a new closure means suitable for clinical practice should be further examined. Fourth, a newly-developed suturing system, OverStitch (Apollo Endosurgery, Austin, TX, United States), was not studied because the evidence in regard to human post-EFTR defect closure was still limited, consisting of a few case series [30] and case reports only. Thus, the popular closure methods including PSS and OTSC were applied in the present study. Fifth, since this study focused on the closure strength on each endoscopic procedure, the variables involving the procedure time of closure, and the completion of inverted closure should be evaluated in the setting of in vivo animal experiences.

## Conclusion

This ex vivo experimental study demonstrated that OTSC closure, which facilitates complete inverted closure, has greater strength than PSS in full-thickness layer suturing. Considering the size limitation suitable for single OTSC, a therapeutic strategy for closing the larger size is further warranted. A clinical study is required to determine whether the basic data obtained in this study are associated with post-EFTR leakage.

## Data Availability

All data generated or analysed during this study are included in this published article.

## Abbreviations

GISTs: Gastrointestinal stromal tumors
EFTR: Endoscopic full-thickness resection
PSS: Purse-string suture
OTSC: Over-the-scope clip
TG forceps: Twin Grasper forceps
SD: Standard deviation
NOTES: Natural orifice transluminal endoscopic surgery

## References

[1] Rubin BP, Heinrich MC, Corless CL (2007). Gastrointestinal stromal tumour. Lancet.

[2] Søreide K, Sandvik OM, Søreide JA, Giljaca V, Jureckova A, Bulusu VR (2016). Global epidemiology of gastrointestinal stromal tumours (GIST): a systematic review of population-based cohort studies. Cancer Epidemiol.

[3] Wilms C, Be T, Early F, Stages WT (2018) Wilms tumor early detection diagnosis and staging can wilms tumors be found early ? Am Cancer Soc. 1–15

[4] Grignani G, Boccone P, Varetto T, Cirillo S (2012). Gastrointestinal stromal tumors. Imaging Tumor Response Ther.

[5] Hiki N, Yamamoto Y, Fukunaga T, Yamaguchi T, Nunobe S, Tokunaga M, Miki A, Ohyama S, Seto Y (2008). Laparoscopic and endoscopic cooperative surgery for gastrointestinal stromal tumor dissection. Surg Endosc Other Interv Tech.

[6] Matsuda T, Nunobe S, Kosuga T, Kawahira H, Inaki N, Kitashiro S, Abe N, Miyashiro I, Nagao S, Nishizaki M, Hiki N (2017). Laparoscopic and luminal endoscopic cooperative surgery can be a standard treatment for submucosal tumors of the stomach: a retrospective multicenter study. Endoscopy.

[7] Suzuki H, Ikeda K (2001). Endoscopic mucosal resection and full thickness resection with complete defect closure for early gastrointestinal malignancies. Endoscopy.

[8] Abe N, Takeuchi H, Ohki A, Hashimoto Y, Mori T, Sugiyama M (2018). Comparison between endoscopic and laparoscopic removal of gastric submucosal tumor. Dig Endosc.

[9] Liu S, Zhou X, Yao YX, Shi K, Yu M, Ji F (2020). Resection of the gastric submucosal tumor (G-SMT) originating from the muscularis propria layer: comparison of efficacy, patients’ tolerability, and clinical outcomes between endoscopic full-thickness resection and surgical resection. Surg Endosc.

[10] Waseda Y, Doyama H, Inaki N, Nakanishi H, Yoshida N, Tsuji S, Takemura K, Yamada S, Okada T (2014). Does laparoscopic and endoscopic cooperative surgery for gastric submucosal tumors preserve residual gastric motility? Results of a retrospective single-center study. PLoS ONE.

[11] Granata A, Martino A, Amata M, Ligresti D, Tuzzolino F, Traina M (2020). Efficacy and safety of gastric exposed endoscopic full-thickness resection without laparoscopic assistance: a systematic review. Endosc Int Open.

[12] Wang C, Gao Z, Shen K, Cao J, Shen Z, Jiang K, Wang S, Ye Y (2020). Safety and efficiency of endoscopic resection versus laparoscopic resection in gastric gastrointestinal stromal tumours: a systematic review and meta-analysis. Eur J Surg Oncol.

[13] Aslanian HR, Sethi A, Bhutani MS, Goodman AJ, Krishnan K, Lichtenstein DR, Melson J, Navaneethan U, Pannala R, Parsi MA, Schulman AR, Sullivan SA, Thosani N, Trikudanathan G, Trindade AJ, Watson RR, Maple JT (2019). ASGE guideline for endoscopic full-thickness resection and submucosal tunnel endoscopic resection. VideoGIE.

[14] Zhou PH, Yao LQ, Qin XY, Cai MY, Xu MD, Zhong YS, Chen WF, Zhang YQ, Qin WZ, Hu JW, Liu JZ (2011). Endoscopic full-thickness resection without laparoscopic assistance for gastric submucosal tumors originated from the muscularis propria. Surg Endosc.

[15] Shi Q, Chen T, Zhong YS, Zhou PH, Ren Z, Xu MD, Yao LQ (2013). Complete closure of large gastric defects after endoscopic full-thickness resection, using endoloop and metallic clip interrupted suture. Endoscopy.

[16] Guo J, Liu Z, Sun S, Liu X, Wang S, Ge N, Wang G, Qi Y (2015). Endoscopic full-thickness resection with defect closure using an over-the-scope clip for gastric subepithelial tumors originating from the muscularis propria. Surg Endosc.

[17] Kobara H, Mori H, Nishiyama N, Fujihara S, Okano K, Suzuki Y, Masaki T (2019). Over-the-scope clip system: a review of 1517 cases over 9 years. J Gastroenterol Hepatol.

[18] Matthes K, Jung Y, Kato M, Gromski MA, Chuttani R (2011). Efficacy of full-thickness GI perforation closure with a novel over-the-scope clip application device: an animal study. Gastrointest Endosc.

[19] Gonzalez J-M, Saito K, Kang C, Gromski M, Sawhney M, Chuttani R, Matthes K (2015). Prospective randomized comparison of gastrotomy closure associating tunnel access and over-the-scope clip (OTSC) with two other methods in an experimental ex vivo setting. Endosc Int Open.

[20] Parodi A, Repici A, Pedroni A, Blanchi S, Conio M (2010). Endoscopic management of GI perforations with a new over-the-scope clip device (with videos). Gastrointest Endosc.

[21] Kobara H, Nishiyama N, Fujihara S, Tada N, Kozuka K, Matsui T, Takata T, Chiyo T, Kobayashi N, Fujita K, Yachida T, Okano K, Suzuki Y, Nishiyama A, Mori H, Masaki T (2021). Traction-assisted endoscopic full-thickness resection followed by O-ring and over-the-scope clip closure in the stomach: an animal experimental study. Endosc Int Open.

[22] Granata A, Martino A, Ligresti D, Zito FP, Amata M, Lombardi G, Traina M (2021). Closure techniques in exposed endoscopic full-thickness resection: overview and future perspectives in the endoscopic suturing era. World J Gastrointest Surg.

[23] Turnbull D, Webber S, Hamnegard CH, Mills GH (2007). Intra-abdominal pressure measurement: validation of intragastric pressure as a measure of intra-abdominal pressure. Br J Anaesth.

[24] Janssen P, Verschueren S, Giao Ly H, Vos R, Van Oudenhove L, Tack J (2011). Intragastric pressure during food intake: a physiological and minimally invasive method to assess gastric accommodation. Neurogastroenterol Motil.

[25] El-Serag HB, Tran T, Richardson PEG (2006). Anthropometric correlates of intragastric pressure. Scand J Gastroenterol.

[26] Iqbal A, Haider M, Stadlhuber RJ, Karu A, Corkill S, Filipi CJ (2008). A study of intragastric and intravesicular pressure changes during rest, coughing, weight lifting, retching, and vomiting. Surg Endosc Other Interv Tech.

[27] Ego M, Abe S, Nonaka S, Suzuki H, Yoshinaga S, Oda I, Saito Y (2021). Endoscopic closure utilizing endoloop and endoclips after gastric endoscopic submucosal dissection for patients on antithrombotic therapy. Dig Dis Sci.

[28] Tera HAC (1976). Tissue holding power to a single suture in different parts of the alimentary tract. Acta Chir Scand.

[29] Yeginsu A, Ergin M, Erkorkmaz U (2007). Strength of esophageal closure techniques with and without tissue reinforcement. World J Surg.

[30] Andalib I, Yeoun D, Reddy R, Xie S, Iqbal S (2018). Endoscopic resection of gastric gastrointestinal stromal tumors originating from the muscularis propria layer in North America: methods and feasibility data. Surg Endosc.

